# Efficacy of spironolactone in treating hidradenitis suppurativa in women of childbearing age: a single-center retrospective analysis

**DOI:** 10.1097/JW9.0000000000000159

**Published:** 2024-07-01

**Authors:** Suma V. Gangidi, Rachel K. Greene, Eric Olsen, Shanelle Jackson, Mio Nakamura

**Affiliations:** a Department of Clinical Sciences, Carle Illinois College of Medicine, University of Illinois Urbana-Champaign, Urbana, Illinois; b Department of Dermatology, University of Michigan, Ann Arbor, Michigan; c Department of Medicine, Wellstar Health System, Kennestone Hospital Graduate Medical Education, Marietta, Georgia; d Department of Medicine, Michigan State University College of Human Medicine, Grand Rapids, Michigan; e Department of Dermatology, University of Michigan, Ann Arbor, Michigan

**Keywords:** antiandrogen, hidradenitis suppurativa, spironolactone, women of childbearing age

## Abstract

**Background:**

Hidradenitis suppurativa (HS) is a chronic skin disease characterized by recurrent nodules that affect areas with a high density of apocrine sweat glands, such as the axillae and groin. Androgens are implicated in the pathophysiology of HS. Therefore, spironolactone, an antiandrogen therapy, is recommended. However, data on its use in women of childbearing age are limited, especially since its antiandrogenic effects may affect menstruation, fertility, and pubertal development.

**Objective:**

To evaluate the efficacy and safety of spironolactone in the treatment of hidradenitis suppurativa in women of childbearing age and to identify factors associated with treatment response.

**Methods:**

A retrospective analysis was conducted on female patients aged 12 to 50 with HS treated with spironolactone at Michigan Medicine dermatology clinics from 2000 to 2021. The patients’ demographic data, HS characteristics, and spironolactone responses were examined. Statistical assessments were performed to determine the efficacy indicators.

**Results:**

Of the 157 patients reviewed, 31 showed an improvement in treatment. Variables such as axillary involvement, previous treatment failures, and use of intralesional steroids were linked to a lack of improvement in spironolactone. Through adjusted multiple logistic regression analysis, a significant association was observed between improvement status and Hurley stage 3 (odds ratio = 0.15 [95% CI: 0.02–0.79], *P* = .036), suggesting that patients with Hurley stage 3 were 85% less likely to exhibit improvement in spironolactone therapy.

**Limitations:**

The study’s retrospective nature and reliance on single-center data can limit generalizability. The sample size is limited and therefore affects the study’s statistical power.

**Conclusion:**

Thus, spironolactone may offer therapeutic benefits for HS in women of childbearing age. However, patients with severe disease (Hurley stage 3) had reduced response rates. Further prospective studies are recommended to validate these findings and determine the most suitable patient profile for spironolactone therapy for HS.

**What is known about this subject in regard to women and their families?**

- Hidradenitis suppurativa (HS) is a chronic skin condition that often affects women, with a greater prevalence in women of childbearing age.
- Traditionally, antibiotics, corticosteroids, biologics, and surgery have been the mainstays for therapy; however, many patients continue to experience recurrent disease.

**What is new from this article as messages for women and their families?**

- Spironolactone may be an effective adjunct therapy in mild cases of HS, especially if initiated early.
- Spironolactone may be less effective in patients with severe or long-standing diseases.

## Introduction

Hidradenitis suppurativa (HS) is a chronic inflammatory skin disease characterized by recurrent painful nodules, sinus tracts, fistulas, and scarring that typically affects areas with a high density of apocrine sweat glands, such as the axillae, groin, and breasts.^1^ This disease predominantly affects women and is 3 times more common in women than in men. While HS affects women of all ages, it is more prevalent among women of childbearing age (WOCA).^2–4^

Given the typical emergence of the disease after puberty, it is suspected that hormones, particularly androgens, play an integral role in the development of HS.^5^ Conditions such as polycystic ovarian syndrome (PCOS), in which the ovaries secrete abnormal androgen levels, are also associated with HS.^6^ Androgens are critical stimulants of follicular keratinocyte proliferation, resulting in obstruction and nodule formation.^7^ Apart from hormonal influences, genetic factors have also been implicated, with studies suggesting that up to one-third of the patients with HS have a family history of the disease.^8^ Environmental factors, such as a history of smoking, obesity, and stress, have been identified as triggers for HS.^4^

Various treatment modalities exist for HS, including antibiotics, corticosteroids, surgery, and biological agents, and have shown varying degrees of success.^9^ Spironolactone, a drug with antiandrogenic properties,^10^ has also shown promising results in several studies.^11,12^ However, literature is sparse regarding the use of spironolactone in WOCA, even though they make up a significant portion of HS patients. It is imperative to investigate the efficacy of spironolactone in this population.

This study aimed to retrospectively investigate the efficacy of spironolactone in treating HS in postpubertal and premenopausal women. The findings will contribute significantly to the understanding of the therapeutic potential of spironolactone in the management of HS in WOCA patients.

## Methods

This was a single-center retrospective cohort study of female patients of childbearing age with HS treated with spironolactone. A medical records search database was used to identify patients diagnosed with HS at the outpatient dermatology clinics of Michigan Medicine, a large tertiary referral center, between 2000 and 2021. The HS diagnosis was determined based on the International Classification of Diseases, Tenth Revision (ICD-10) diagnostic code L73.2 and ICD-9 code 705.83. Exclusion criteria included the use of spironolactone for less than 3 months, based on studies showing that it takes 3 to 6 months of treatment with spironolactone for clinical improvement.^10^

Information extracted from each patient’s medical records included demographic details (female sex, age, and race/ethnicity) and insurance status (private, US Military, Medicaid, and Medicare). The characteristics of HS were collected, including confirmation of HS diagnosis (clinically and/or histologically), age at diagnosis, location(s) of affected areas, Hurley stage, and association of flares with menses. Clinical notes were analyzed to confirm spironolactone use, dosing, duration, and response. Additional variables collected included Fitzpatrick skin type (FST), HS family history, and comorbidities, such as obesity, acne, and PCOS.

Improvement status was classified based on objective clinician assessments and organized into 2 categories: improvement and no improvement. Clinician assessments involve a thorough review of medical records, physical examinations, and an evaluation of treatment response-based patients’ assessment of symptoms, pain, and lesion count. The classification of “improving” was based on documented reductions in lesion count, pain, and symptoms. The classification of “no improvement” was determined by increased lesion count, pain, or clinical indicators of HS progression. Additional considerations for efficacy included the addition of concomitant therapies or a change to more aggressive therapies, as well as physician-noted effectiveness indicators (including suggestions for improvement, stability, flare-ups, and worsening).

### Statistical analysis

Statistical analysis was performed using R software (version 4.1.1; R Core Development Team, Vienna, Austria) and RStudio (version 1.1.1717; R Studio Inc., Boston, MA). Data were summarized using descriptive statistics, with median and interquartile ranges (IQRs) for continuous variables and frequencies and proportions for categorical variables. Comparisons between groups were conducted using the Mann–Whitney parametric test for continuous variables and the Fisher exact test for categorical variables.

Bivariate analyses were conducted to investigate the specific associations within the cohort. Chi-squared tests were used to examine these relationships. All tests were 2-tailed, and a *P* value less than .1 was considered statistically significant.

An adjusted multiple logistic regression model was used to assess the association between the improvement status of spironolactone and various factors. Variables were included in the model based on unadjusted *P* values and known associations with HS treatment outcomes (in the case of acne and obesity). Potential multicollinearity was examined using variance inflation factors, and variables contributing to multicollinearity were excluded, including cotreatment with metformin and intralesional steroids. Model selection for the multiple logistic regression model was further guided by the Akaike Information Criterion, with lower Akaike Information Criterion values indicating a better fit.

## Results

### Demographics

We identified 157 female patients with HS between 12 and 50 years treated with spironolactone for at least 3 months. Demographic information is shown in Table 1. The median age of our cohort was 36.5 years, with an IQR of 10.4 years. The median age of diagnosis was 22.8 years, with an IQR of 10.3 years. Most patients were identified as non-Hispanic (*n* = 149, 96%), with the highest representation of White individuals (*n* = 91, 59%), followed by Black individuals (*n* = 56, 36%). The most common FST was II (*n* = 42, 32%), followed by I (*n* = 36, 27%). FST data were unavailable for a subset of patients (*n* = 24, 15.3%). Patients most commonly presented with Hurley stage 2 disease (*n* = 66, 43%), followed by stage 3 (*n* = 44, 29%). The axilla was the most affected location (*n* = 126, 80%), followed by the groin (*n* = 107, 68%), inframammary area (*n* = 73, 46%), buttocks (*n* = 41, 26%), thighs (*n* = 21, 13%), and abdomen (*n* = 14, 8.9%). Of the 119 patients with available information, half (*n* = 60, 50%) reported an association of HS flare with their menstrual cycle. Of 142 patients with available information, PCOS was present in 47 (33%) patients. A family history of HS was reported in 13 patients (23%).

**Table 1:** Baseline demographics and characteristics

Characteristic	No improvement, *n* = 126^a^	Improvement, *n* = 31^a^	Overall, *N* = 157^a^	*P* value^b^
Age of diagnosis, median (IQR)	18.50 (17.25)	21.50 (15.00)	22.81 (10.33)	.88
Ethnicity: non-Hispanic, *n* (%)	120 (97%)	29 (94%)	149/155 (96%)	.34
Race, *n* (%)				.76
Caucasian	72 (59%)	19 (61%)	91/154 (59%)	
Black/African American	45 (37%)	11 (35%)	56/154 (36%)	
Asian	3 (2.4%)	0 (0%)	3/154 (1.9%)	
Multiracial	1 (0.8%)	1 (3.2%)	2/154 (1.3%)	
American Indian/Alaska	1 (0.8%)	0 (0%)	1/154 (0.6%)	
Other	1 (0.8%)	0 (0%)	1/154 (0.6%)	
Fitzpatrick skin type, *n* (%)				.40
I	29 (27%)	7 (27%)	36/133 (27%)	
II	34 (32%)	8 (31%)	42/133 (32%)	
III	4 (3.7%)	3 (12%)	7/133 (5.3%)	
IV	9 (8.4%)	4 (15%)	13/133 (9.8%)	
V	28 (26%)	4 (15%)	32/133 (24%)	
VI	3 (2.8%)	0 (0%)	3/133 (2.3%)	
Hurley stage, *n* (%)				.068
1	29 (24%)	13 (43%)	42/152 (28%)	
2	54 (44%)	12 (40%)	66/152 (43%)	
3	39 (32%)	5 (17%)	44/152 (29%)	
Family history of HS, *n* (%)	9 (20%)	4 (36%)	13/56 (23%)	.26
HS flare associated with menses (yes/no), *n* (%)	43 (47%)	17 (63%)	60/119 (50%)	.14
History of PCOS? (yes/no), *n* (%)	40 (35%)	7 (24%)	47/142 (33%)	.25
Obesity, *n* (%)	101 (82%)	23 (74%)	124/154 (81%)	.32
Acne, *n* (%)	79 (66%)	19 (61%)	98/151 (65%)	.64
Locations affected, *n* (%)				
Axilla	107 (85%)	19 (61%)	126/157 (80%)	.003
Groin	87 (69%)	20 (65%)	107/157 (68%)	.63
Thigh	18 (14%)	3 (9.7%)	21/157 (13%)	.77
Buttocks	33 (26%)	8 (26%)	41/157 (26%)	.97
Abdomen	12 (9.5%)	2 (6.5%)	14/157 (8.9%)	.74
Inframammary	57 (45%)	16 (52%)	73/157 (46%)	.52

a Median (IQR); *n* (%).

b Wilcoxon rank sum test; Fisher exact test; Pearson chi-squared test.

HS, hidradenitis suppurativa; IQR, interquartile range; PCOS, polycystic ovarian syndrome.

### Comorbidities

Most of our cohort had a history of obesity (*n* = 124, 81%) and acne (*n* = 98, 65%). Patients who were obese tended to be older (*P* = .053) and more likely to have a family history of HS (*P* = .035). These patients were also more likely to develop inframammary HS (*P* = .065) and PCOS (*P* = .015). Patients diagnosed with acne demonstrated a higher propensity for axillary HS (*P* = .061) and were more likely to experience HS flares associated with their menstrual cycles (*P* < .001). Notably, patients reporting HS flares with menses were, on average, younger (*P* = .025) and tended to receive a lower dose of spironolactone (*P* < .065).

### Concomitant therapy

Patients on biological therapies were typically older (*P* = .044), had a higher likelihood of HS in the buttocks (*P* < .001), and presented with HS in 3 or more locations (*P* < .001). These patients were also more likely to have Hurley stage 3 HS (*P* < .001) and to be on higher doses of spironolactone (*P* < .001). The use of concurrent topical steroids (*P* = .004) and topical antibiotics (*P* = .003) was also higher in this group, with a significant number of patients having previously failed the treatment (*P* < .048). Patients taking metformin also tended to have higher doses of spironolactone (*P* < .001).

### Spironolactone treatment

The median prescribed dose of spironolactone was 100 mg daily, with the most common dose being 50 to 100 mg daily. The median time to start spironolactone was 8.8 years after the date of HS diagnosis.

Patients were divided into 2 groups: those who experienced improvement in spironolactone (*n* = 31, 20%) and those who did not (*n* = 126, 80%). The associations between therapeutic responses and patient characteristics are shown in Tables 1 and 2. There were no significant differences in age, sex, ethnicity, race, insurance type, FST, family history of HS, obesity, or acne between the 2 groups. A shorter duration between the diagnosis and initiation of spironolactone was associated with improvement in spironolactone (*P* = .047). Involvement of the axilla (*P* = .003) and use of intralesional steroids (*P* = .015) were associated with a lack of improvement in spironolactone. Additionally, patients who had received other treatments before spironolactone (*P* = .023) and those who had failed previous treatments (*P* = .030) showed no improvement with spironolactone. A higher proportion of patients with Hurley stage 1 disease showed improvement with spironolactone compared with stages 2 and 3 (*P* = .068). Lastly, 4 patients with no improvement on spironolactone experienced improvement with surgical intervention.

**Table 2:** Summary of spironolactone treatment characteristics based on improvement status

Characteristic	No improvement, *n* = 126^a^	Improvement, *n* = 31^a^	Overall, *N* = 157^a^	*P* value^b^
Spironolactone start year after diagnosis, median (IQR)	7.00 (10.25)	3.00 (9)	8.80 (8.93)	**.047**
Received other treatment before spironolactone, *n* (%)	114 (94%)	24 (80%)	138/151 (91%)	**.023**
Failed previous treatment, *n* (%)	114 (90%)	23 (74%)	137/157 (87%)	**.030**
Highest dose of spironolactone (mg), *n* (%)				.89
>200	1 (0.8%)	0 (0%)	1/157 (0.6%)	
>150 and ≤200	23 (18%)	7 (23%)	30/157 (19%)	
>100 and ≤150	14 (11%)	4 (13%)	18/157(11%)	
>50 and ≤100	51 (40%)	13 (42%)	64/157(41%)	
≤50	37 (29%)	7 (23%)	44(28%)	
Other concomitant therapy, *n* (%)				
Retinoids (isotretinoin)	1 (0.8%)	1 (3.2%)	2/157 (1.3%)	.4
Systemic steroid	9 (7.1%)	0 (0%)	9/157 (5.7%)	.2
Topical steroid	6 (4.8%)	2 (6.5%)	8/157 (5.1%)	.7
Intralesional steroid	28 (22%)	1 (3.2%)	29/157 (18%)	**.015**
Metformin	13 (10%)	0 (0%)	13/157 (8.3%)	.073
Zinc	10 (7.9%)	2 (6.5%)	12/157 (7.6%)	>.9
Biologic	34 (27%)	6 (19%)	40/157 (25%)	.4
Humira (adalimumab), *n* (%)	25 (20%)	4 (13%)	29/157 (18%)	.37
Stelara (ustekinumab), *n* (%)	6 (4.8%)	0 (0%)	6/157 (3.8%)	.60
Remicade (infliximab), *n* (%)	3 (2.4%)	1 (3.2%)	4/157 (2.5%)	>.99
Other, *n* (%)	3 (3.4%)	1 (3.2%)	4/157 (2.5%)	>.99
Oral contraceptives (OCPs)	6 (4.8%)	0 (0%)	6/157 (3.8%)	.6
Oral antibiotic(s)	65 (52%)	16 (52%)	81/157 (52%)	>.9
Topical antibiotic	89 (71%)	21 (68%)	110/157 (70%)	.8
Topical wash	115 (91%)	24 (77%)	139/157 (89%)	**.053**

Bold-faced *P* values denote significance.

a Median (IQR); *n* (%).

b Wilcoxon rank sum test; Fisher exact test; Pearson Chi-squared test.

IQR, interquartile range.

Using this data, an adjusted multiple logistic regression analysis (Table 3) assessed the association between the improvement status of spironolactone and various factors. No significant associations were found between improvement status and year of diagnosis, obesity, acne, HS flare associated with menses, Hurley stage 2, highest dose of spironolactone, biologic use, topical wash use, receiving other treatments before spironolactone, axillary association, and failure of other treatments. However, a significant association was observed between improvement status and Hurley stage 3 (odds ratio = 0.15 [95% confidence interval: 0.02–0.79], *P* = .036), suggesting that patients with Hurley stage 3 were 85% less likely to exhibit improvement with spironolactone therapy.

**Table 3:** Adjusted multiple logistic regression analysis assessing the association between improvement status on spironolactone

Variable	Odds ratio [95% CI]	*P*(> *z* )
Start year after diagnosis	0.95 [0.88–1.01]	.11
Obesity	0.97 [0.25–4.35]	>.9
Acne	1.34 [0.34–5.88]	.7
Axilla	0.24 [0.05–1.14]	**.072**
HS flare associated with menses (yes/no)	2.31 [0.71–8.06]	.2
Hurley stage 2	0.44 [0.12–1.64]	.2
Hurley stage 3	0.15 [0.02–0.79]	**.036**
Highest dose of spironolactone (mg)	1.00 [0.99–1.01]	.6
Biologic	1.53 [0.31–7.13]	.6
Topical wash	0.47 [0.07–3.27]	.4
Received other treatment before spironolactone	0.63 [0.02–15.7]	.8
Failed treatment	2.63 [0.15–85.0]	.5

Bold values denote statistical significance.

CI, confidence interval; HS, hidradenitis suppurativa.

## Discussion

The primary aim of our study was to understand the efficacy of spironolactone in treating WOCA with HS. We also aimed to understand patient characteristics and clinical features that may be associated with response to spironolactone therapy. To our knowledge, this is the largest retrospective study reported in the literature to date to investigate the use of spironolactone for managing HS.

Our cohort included 157 WOCA and HS patients treated with spironolactone for at least 3 months. Most patients presented with Hurley stage 2 disease, suggesting a predominance of moderate disease severity in our cohort. The most common location of HS involvement is the axilla. Most patients were obese, over half had acne, and approximately one-third had PCOS. Approximately half of the patients reported HS flares during their menstrual cycle. These findings reflect the possible role of hormonal fluctuations or imbalances in HS.

Although spironolactone is the recommended treatment for HS, a large proportion of our cohort did not exhibit marked improvement with this therapy. We found that only 20% responded positively to a median spironolactone dose of 100 mg daily. This finding may support the concept that an androgen-mediated HS phenotype may exist,^13^ similar to the androgen mediation observed in PCOS.^14^ Similarly, previous studies noted no significant change in Hurley stage or fistula count following treatment with spironolactone.^12^ However, a study by Lee and Fisher^15^ found that 85% of patients were highly responsive to spironolactone 100 mg daily, with most achieving remission within 3 to 4 months of initiation. However, this study was limited by the small sample size of 20 patients with HS involving only the groin. Patients were also mostly White (85%) and were less likely to be obese compared to our cohort (40% vs 81%), and spironolactone was initiated as the initial treatment. Some studies also suggest that spironolactone may improve symptoms in patients with HS, demonstrating an improved response to antibiotics and improvement in pain.^12,16^

Patient characteristics such as age, race, and ethnicity were unrelated to spironolactone response. Similarly, Toker et al.^17^ found that race, ethnicity, and FST may not influence HS disease burden even after accounting for known disease parameters. We found that patients with Hurley stage 1 disease were more responsive to spironolactone therapy than those with stages 2 or 3 disease, and patients with Hurley stage 3 disease were 85% less likely to exhibit improvement with spironolactone therapy. Improvement in spironolactone was also associated with a shorter time from diagnosis to spironolactone initiation.

A history of other treatments and previous unsuccessful therapies correlated with a poorer response to spironolactone. Our findings suggest that spironolactone may be less effective in patients with severe or long-standing diseases. Spironolactone monotherapy may not be a practical therapeutic strategy for patients who have failed other treatment options, such as systemic antibiotics.^18^ Spironolactone may be beneficial if implemented as primary or ancillary treatment in patients with mild disease. This finding mirrors the suggestion of McPhie et al.^19^ that spironolactone may be an appropriate agent for treating mild disease.

Our results showed that the 4 patients with no improvement on spironolactone experienced improvement with surgical intervention. The recurrence rate following surgery can range from 6 to 37%, depending on the type of procedure performed and the location of the lesion.^20^ Given the small sample size, we could not identify any significant association. Further research on this topic could help highlight the importance of integrating surgical options into a broader therapeutic framework of treatment-refractory HS.

Our study had some notable limitations. First, the retrospective nature of this study poses inherent biases, with the possibility of missing or misclassified data from patient records. The reliance on ICD-9 and ICD-10 codes is subject to misclassification and may not provide the full spectrum of HS patients, such as those with syndromes such as PASH (pyoderma gangrenosum, acne, and HS) and PAPASH (pyogenic arthritis, pyoderma gangrenosum, acne, and HS). The single-center nature of the study limits the generalizability of our findings due to the region-specific patient populations. There was an absence of standardized objective measures with treatment improvement based on reporting by clinicians, which introduced variability in the interpretation of treatment outcomes. Confounding variables, such as concomitant treatment for HS, could not be avoided. Regarding statistical methods, the small sample size and potential multicollinearity in the multiple regression model could have affected the study’s overall findings. Considering these limitations, future research should focus on a multicenter, prospective design and a longer observation period to evaluate clinical improvement. Furthermore, given that HS disproportionately impacts WOCA, it is vital to consider the hormonal influences of spironolactone therapy; further studies evaluating the safety of spironolactone in this patient population, including its effects on fertility, are needed.

## Conclusion

We explored the therapeutic efficacy of spironolactone in WOCA with HS and found that it may be beneficial for patients with mild HS, notably those at Hurley stage 1 if implemented early as a primary or ancillary treatment. Its efficacy is limited to patients with severe, recalcitrant, or long-standing disease. Given the limitations of this single-center retrospective study, there is a need for more expansive, prospective, multicenter studies to elucidate further the safety and efficacy of spironolactone for treating HS.

## Conflicts of interest

The authors made the following disclosures: M.N. serves as an investigator for Amgen, Argenx, Boehringer Ingelheim, Bristol Myers Squibb, Pfizer, and Regeneron and receives research support from Regeneron. M.N. has served on the advisory board for Argenx, Boehringer Ingelheim, and Bristol Myers Squibb. For the other authors, there is no conflicts of interest.

## Funding

None.

## Study approval

The author(s) confirm that any aspect of the work covered in this manuscript that has involved human patients has been conducted with the ethical approval of all relevant bodies.

## Author contributions

SVG and MN contributed to the study's conceptualization, methodology, and research design. MN was additionally involved with project supervision and editing the manuscript. SVG, RKG, and EO were involved in the investigation, data curation, writing, and manuscript editing. SJ contributed to the conceptualization, writing, and editing of the manuscript.
